# Community health worker roles in intervention delivery: a scoping review of heart disease and stroke prevention trials in the United States

**DOI:** 10.3389/fstro.2025.1658612

**Published:** 2025-10-02

**Authors:** Imama A. Naqvi, Clare C. Bassile, S. Reza Ebadi, Dakembay E. Hoyte, Lauren N. Paguirigan, Juan Meyreles, Glenn McMillan, Ian M. Kronish, Olajide A. Williams

**Affiliations:** ^1^Department of Neurology, Columbia University Medical Center, New York, NY, United States; ^2^Department of Rehabilitation & Regenerative Medicine, Programs in Physical Therapy, Columbia University Medical Center, New York, NY, United States; ^3^Department of Medicine, Center for Behavioral Cardiovascular Health, Columbia University Medical Center, New York, NY, United States

**Keywords:** community health worker, preventive trials, health promotion, stroke prevention, cardiovascular disease prevention

## Abstract

Heart disease (HD) and stroke risk can be reduced with adequate cardiovascular disease (CVD) disease prevention as outlined in the American Heart Association's Life's Essential 8 guidelines for modifiable health behaviors. This scoping review examines the roles of community health workers (CHWs) in CVD prevention trials across the United States. In the 24 clinical trials identified, our review emphasizes the effectiveness of CHWs in improving health behaviors and outcomes, particularly for underserved populations with limited access to health care. CHWs were actively engaged in implementing interventions, providing culturally sensitive education, offering health coaching, and supporting lifestyle modifications, such as increased physical activity and medication compliance. Notably, while most studies focused on HD, only three specifically targeted secondary stroke prevention. Beyond their role of delivering behavioral interventions, CHWs supported research efforts by collecting data and maintaining participant involvement. However, their integration into academic teams was inconsistent in terms of scope of practice and level of interprofessional engagement. Furthermore, CHW research contributions were rarely recognized, with a handful acknowledged in publications. Training for CHWs generally included disease-specific knowledge and communication skills. CHW training programs varied considerably in their scope and standards, with unclear role definitions and insufficient collaboration with academic institutions. To enhance CHW-led preventive health care, developing standardized training frameworks, defining CHW responsibilities in clinical and research collaborations and building sustainable community–academic partnerships are suggested. These actions could significantly increase CHWs' role in reducing CVD disparities, thereby promoting more equitable health care across the United States.

## Introduction

Cardiovascular disease (CVD), including heart disease (HD) and stroke, remains the leading cause of the global burden of death and disability. This is despite the existence of medical and behavioral strategies that can effectively prevent these cardiovascular events (Martin et al., 2024). The American Heart Association's (AHA) Life's Essential 8 identifies cardiovascular health as driven by key health behaviors of physical activity (PA), nutritious diet, smoking cessation, sufficient sleep, and control of blood pressure, cholesterol, blood sugar, and weight—as vital for preventing CVD (Lloyd-Jones et al., 2022). The burden falls heavily on underserved populations, particularly in low- and middle-income countries (LMICs), which bear more than 80% of stroke-related mortality (Feigin et al., 2021). In high-income settings such as the United States, mortality rates are higher among minoritized communities (Furie, 2020) who face heightened risks due to socioeconomic challenges (Willey et al., 2011). Primary prevention relying on lifestyle changes and medications and secondary prevention after a cardiovascular event using antiplatelet therapy and stricter risk factor management (Boehme et al., 2017) are critical yet difficult to implement in underserved areas due to limited access to health care systems and competing demands that interfere with individual-level health behaviors (Kernan et al., 2014). Furthermore, tertiary prevention to improve health outcomes and reduce disability demands continuous care and rehabilitative services that are often limited in under-resourced communities (Winstein et al., 2016).

Community health workers (CHWs) are public health professionals who are rooted in their communities and use trust and cultural understanding to enhance health care delivery in underserved regions (Rosenthal et al., 2010). They can bridge community and health services to promote health care delivery (Spencer et al., 2010). With a synchronous lens of cultural competency, they can enhance health literacy and reduce health inequities in minoritized populations [American Public Health Association (APHA), 2009]. A systematic review found that CHWs are particularly effective in disease prevention by enhancing knowledge, supporting lifestyle adherence, and improving access to care, thereby supporting both CVD prevention and recovery (Viswanathan et al., 2010).

Although CHWs are increasingly integral to community-based CVD interventions, uncertainties persist about the scope of training needed and the level of integration into team roles. This is important as it directly affects the quality of intervention delivery with respect to the resources allocated for community training and implementation fidelity. While traditional roles include serving as community mediators in improving culturally appropriate education, resources, or direct services, they can also engage as community organizers in leadership development and capacity-building projects (Spencer et al., 2010) and have even directly delivered CVD interventions in LMICs (Irvin and Sentell, 2019). In the United States, where health care is privatized and resources are concentrated in large academic institutions with multilevel health-systems barriers, minoritized communities stand to gain the most support from strategically implemented CHW-led interventions [Spencer et al., 2010; American Public Health Association (APHA), 2009]. This scoping review examines CVD prevention trials conducted in the United States to guide equitable strategies that can maximize CHWs' role and sustained impact.

We sought to (1) outline CHW roles in CVD prevention trials, separating their delivery of community-based interventions (traditional roles, e.g., health coaching) and participation in research related roles, for example, study documentation; (2) evaluate training approaches for tasks tied to interventions, including those addressing health behaviors; and (3) explore structural and systems-level facilitators and barriers to CHWs' impact among minoritized communities. The findings may inform effective training frameworks, integration methods, and policy recommendations to strengthen CHWs' role in reducing health care disparities.

## Methods

**Figure 1:**
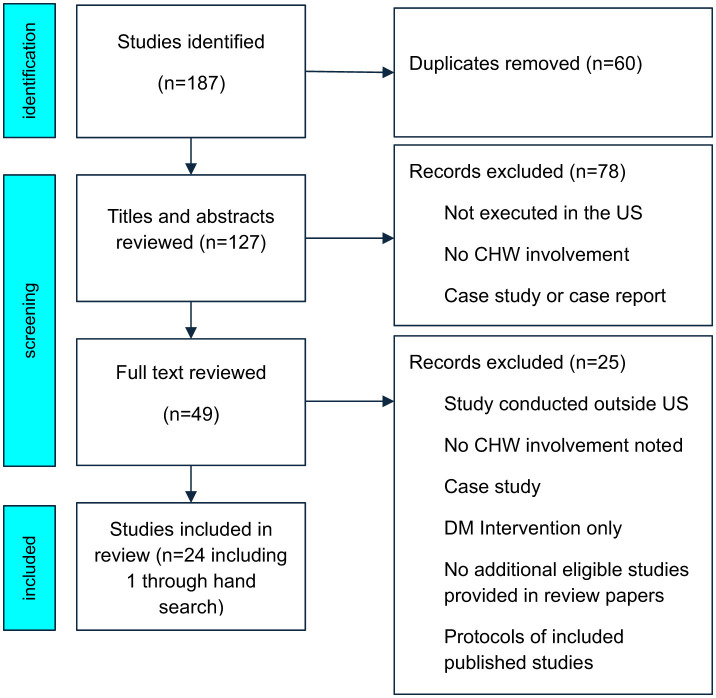
Flow diagram of study selection process. CHW, community health workers; DM, diabetes mellitus.

We chose a scoping review to summarize key concepts and identify gaps in CHW-integrated preventive interventions. We followed the reporting guidelines of Preferred Reporting Items for Systematic reviews and Meta-Analyses Extension for Scoping Reviews (see Figure 1 and the Supplemental material).

### Search strategy

PubMed was the primary database used to search for studies published since the database's inception through October 17, 2024. This was to ensure that we captured the widest audience and the highest impact community-led interventions in the United States. We used all the search terms for CHWs paired with Medical Subject Headings terms for CVD (e.g., coronary artery disease, myocardial infarction, and heart failure) OR cerebrovascular accident OR hypertension OR hyperlipidemia (see the Supplemental material). The studies included all clinical trials, randomized controlled trials, or stand-alone study protocols for trials involving CHWs and interventions to prevent HD or stroke written in English. Systematic/scoping/narrative reviews were originally included to identify additional citations. Articles were excluded if the intervention was not conducted in the United States, if it was a case study or report, if the intervention addressed only individuals with diabetes mellitus, or if the authors did not mention CHW participation.

### Literature selection

A data extraction table that included the following was used to pull the relevant information from each fully reviewed paper: authors, article title, publication year, initial reviewer, confirmation reviewer, include/exclude (yes/no), study type, chronic disease targeted, location of the study, CHW roles, study intervention, study outcomes, CHW barriers and facilitators, CHW training (including didactic and skills content areas, hours, competency evaluation, and supervision procedures), and notes for additional information. Two authors (DH, LP) independently conducted the initial search, reviewed titles and abstracts for eligibility, and came to consensus when there was disagreement. All full-text articles for final inclusion/exclusion decision and data extraction by at least two authors (CCB, SRE, and IAN). If discrepancies occurred, agreement was reached through discussion. The search identified 127 records after duplicates were removed. An article title and abstract review yielded 49 studies of potential relevance, requiring full-text review. A total of 24 studies met final eligibility criteria and were included in this review (Figure 1).

## Results

### General study characteristics

Among the 24 studies, only 3 focused distinctly on secondary and tertiary stroke prevention (see Table 1 and the Supplemental material). Primary outcomes varied, with overlap among studies: 16 studies (Allen et al., 2014; Balcazar et al., 2009; Balcázar et al., 2010; Becker et al., 2005; Commodore-Mensah et al., 2024; Daniels et al., 2012; Ephraim et al., 2014; Heisler et al., 2022; Ibe et al., 2021; Johansson et al., 2023; Katula et al., 2017; Krieger et al., 1999; Levine et al., 2003; Margolius et al., 2012; Shah et al., 2024; Towfighi et al., 2021) mainly aimed to improve blood pressure control, while 12 (Allen et al., 2014; Balcazar et al., 2009; Balcázar et al., 2010; Becker et al., 2005; Ephraim et al., 2014; Johansson et al., 2023; Katula et al., 2017; Krieger et al., 1999; Shah et al., 2024; Towfighi et al., 2021; Islam et al., 2023; Samuel-Hodge et al., 2020) included other risk factor control (see the Supplemental Material).

**Table 1A:** Summary of key studies with CHW roles in cardiovascular disease prevention non-stroke-focused trials.

**Study identifier/study design**	**CHW role type**	**Role definition**	**Training-specific to intervention**	**Delivered interventions**
(Allen et al. 2014) (COACH)/NP/CHW team vs. enhanced usual care + protocol (Allen et al., 2011)	Traditional roles	Health coaching	Didactic: • Lifestyle counseling strategies • Drug adherence counseling • Behavioral goal setting	Reinforced NP instructions on lifestyle and medication adherence; helped develop behavior change plans
		Providing culturally appropriate services	Didactic: • Cultural sensitivity training • Communication bridging strategies	Built rapport through shared background and trust; enhanced communication with underserved patients
		Motivational interviewing	Didactic: • Motivational interviewing principles • Adherence promotion strategies	Provided individualized adherence support using MI techniques; reinforced NP guidance
		Decreasing barriers	Practical: • Evaluation of barriers to adherence • Problem-solving support	Identified barriers (e.g., social, financial), assisted with problem-solving to enhance adherence
	Research roles	Study documentation	Practical: • Documentation practices • Encounter tracking and fidelity monitoring	Recorded patient contact time, content of encounters, and follow-up preparation activities
		Assisted adherence	Practical: • Medication tracking • Appointment reminder techniques	Supported medication and appointment adherence with follow-up calls and logs
(Balcazar et al. 2009) (Promotoras de Salud Contra la Hipertension)/Promotora-led 9-week intervention vs. educational materials only	Traditional roles	Educating	Didactic: • 4-day “Your Heart, Your Life” (NHLBI) training • 6 educational modules on HTN, nutrition, and physical activity	Delivered weekly sessions using SPSC curriculum, covering HTN management, physical activity, diet, weight control, salt/fat reduction
		Health coaching	Practical: • Use of photonovela and action-oriented behavior change • Family-centered lifestyle coaching	Guided participants through realistic vignettes on stress management, doctor visits, medication adherence; facilitated family involvement
		Providing culturally appropriate services	Didactic: • Materials culturally tailored for Mexican Americans • Spanish delivery, bilingual promotoras	All sessions delivered in Spanish by promotoras from participants' community; used culturally familiar stories and materials
	Research roles	Assisted adherence	Practical: • Monitoring behavior changes through follow-up calls • Medication adherence support using behavior modules	Made phone calls between sessions to check on adherence and reinforce healthy behaviors
		Study documentation	Practical: • Attendance tracking • Follow-up documentation for missed sessions and survey completion	Maintained records of participant attendance, rescheduled missed sessions, tracked survey completion, and collected biometric and behavioral data
(Balcázar et al. 2010) (HEART)/Promotora-led 8-week lifestyle program vs. basic educational materials	Traditional roles	Educating	Didactic: • 16–18 h of training on Su Corazón, Su Vida curriculum • Focus on CVD risk factors and behavioral strategies	Delivered weekly 2-h group sessions on heart-healthy behaviors including nutrition, physical activity, stress, weight, and salt/fat intake
		Health coaching	Practical: • Goal setting and action planning • Motivational support and behavioral reinforcement	Encouraged and reviewed action steps during sessions and follow-ups; reinforced lifestyle changes through phone calls and small group sessions
		Providing culturally appropriate services	Didactic: • Bilingual promotores from the community • Spanish-language delivery • Cultural tailoring of materials	Provided culturally relevant, Spanish-language education using promotores from the same community to build trust and relatability
	Research roles	Assisted adherence	Practical: • Behavior tracking between sessions • Coaching on self-monitoring of risk behaviors	Followed up via calls and group sessions to track behavior change and reinforce adherence to heart-healthy practices
		Study documentation	Practical: • Participant screening and eligibility tracking • Documentation of session attendance and follow-up	Maintained logs of participation, conducted post-intervention assessments (clinical and survey), and tracked participant behavior and engagement
(Becker et al. 2005) (community-based care)/CHW/NP team vs. enhanced primary care	Traditional roles	Educating	Didactic: • Counseling on CHD risk factors (lipids, HTN, smoking) • Diet and physical activity education	CHWs provided counseling on lifestyle modification, medication understanding, and use of exercise facilities like YMCA
		Health coaching	Practical: • Action planning and support • Self-monitoring and coaching on diet, smoking, and physical activity	Facilitated structured follow-ups with coaching on behavior change; reinforced goals via in-person and telephone sessions
		Providing access to social services	Practical: • Resource support for medication and transportation • Access to YMCA and pharmacy cards	Connected patients with free pharmacotherapy, YMCA memberships, and systems to reduce barriers to access (e.g., child care, transit)
	Research roles	Assisted adherence	Practical: • Medication adherence support • Counseling on use of BP and lipid-lowering therapy	Helped participants understand and adhere to prescribed medications; monitored pharmacotherapy pickup and participation in exercise
		Study documentation	Practical: • CBC session tracking and clinical logs • Team reviews of participant progress	Documented CBC encounter frequency, participant progress, prescription uptake; participated in bi-monthly team case reviews
(Commodore-Mensah et al. 2024) (LINKED-HEARTS)/LINKED-HEARTS program vs. enhanced usual care + protocol (Commodore-Mensah et al., 2023)	Traditional roles	Educating	Didactic: • Lifestyle counseling using culturally relevant materials • Education on hypertension and diabetes	CHWs provided culturally relevant education on BP, diabetes, and lifestyle changes at patient-preferred locations
		Health coaching	Practical: • Individualized care plan creation • Biweekly coaching on self-management and lifestyle	Developed individualized plans and reinforced adherence through regular follow-ups (telehealth and in-person)
		Providing access to social services	Practical: • Training on addressing social determinants • Resource navigation and referral processes	Linked participants to community services and addressed structural/social barriers through resource referral
	Research roles	Assisted adherence	Practical: • BP device use and HBPM protocol • Telemonitoring via Sphygmo app	Trained participants on HBPM protocol and monitored telehealth data via mobile app
		Contact the primary care team	Practical: • Communication flow between CHW, pharmacist, and providers • Use of Sphygmo clinician portal	Coordinated with providers and pharmacists regarding BP status, medication, and social factors
		Study documentation	Practical: • Documentation of 7- and 30-day BP averages in EMR • Monthly data and encounter tracking	Recorded BP logs, follow-up encounters, and participant progress in EMR and study tracking system
(Daniels et al. 2012) (ABCD)/CHW-led active learning vs. physician lectures	Traditional roles	Educating	Didactic: • ABCD risk factors education (A1c, BP, Cholesterol, Depression) • Food label reading • Medication instructions	Delivered 6-week interactive sessions using culturally tailored materials to teach CVD risk reduction, food labels, and signs of depression
		Health coaching	Practical: • Return demonstration-based teaching • Peer modeling and reinforcement • Motivational encouragement	Modeled positive behaviors and guided self-monitoring activities; available for peer counseling between sessions
		Decreasing barriers	Practical: • Assertiveness skills • Provider communication training • Health system navigation	Supported participants in understanding prescriptions, appointment cards, and how to communicate effectively with providers
	Research roles	Enrollment of subjects	Practical: • Participant recruitment from churches • Screening based on ABCD risk factors	Recruited church/community members through flyers, health fairs, and peer outreach
		Assisted adherence	Practical: • BP/glucose/cholesterol measurement training • Interpretation of readings	Trained participants on how to check and interpret BP, blood glucose, and cholesterol; guided self-management
		Study documentation	Practical: • Consent processes • Recording workshop attendance and outcome surveys	Collected pre/post knowledge and physiological data, documented session progress, and referred results to primary care when needed
(Ell et al. 2017) (AHH)/Promotora-assisted self-care + depression support vs. PCMH usual care + protocol (Ell et al., 2016) (AHH)	Traditional roles	Health coaching	Practical: • Problem-solving framework training • Self-management skill-building • Goal setting and action planning	Promotoras supported development of action plans and behavioral goals related to health, depression, and lifestyle changes
		Providing access to social services	Practical: • Resource navigation strategies • Referral pathways to health and social services	Connected participants with services such as housing, transportation, legal aid, and support groups based on individual needs
		Providing culturally appropriate services	Didactic: • Spanish-language delivery and cultural tailoring • Fotonovelas and low-literacy materials	Used bilingual, culturally attuned content to build trust and overcome stigma around mental health and chronic disease
	Research roles	Assisted adherence	Practical: • Adherence coaching for medication and behavior • Booster sessions to reinforce regimen	Reinforced medication-taking and care plan adherence via one-on-one support and follow-ups at home or by phone
		Study documentation	Practical: • Session tracking and case note documentation • Supervised fidelity reporting and patient referral notes	Logged intervention content, monitored fidelity, and escalated medical/social concerns to care providers as needed
(Ephraim et al. 2014) (ACT)/CHW alone vs. CHW + family communication vs. CHW + problem-solving	Traditional roles	Educating	Didactic: • NHLBI “With Every Heartbeat is Life” manual • Lifestyle education (BP, medication, exercise, nutrition)	Provided culturally tailored education on hypertension and healthy behaviors; reviewed lifestyle concepts during home visits and calls
		Health coaching	Practical: • Behavioral reinforcement techniques • Longitudinal support skills • Goal setting and action planning	Supported participants through goal setting and regular check-ins to encourage sustained self-management behaviors and confidence
		Providing access to social services	Practical: • Resource identification and referral • Navigation training for community/clinic-based support	Linked patients to clinical and community resources to address barriers to BP control (transportation, clinic access, etc.)
	Research roles	Assisted adherence	Practical: • BP cuff training • Reinforcement of self-monitoring behavior	Trained participants on home BP monitoring and reinforced adherence through follow-ups and support
		Contact the primary care team	Practical: • Communication of alerts to providers • Liaison coordination	Communicated high BP readings and access issues to clinic teams; served as communication bridge between patient and care providers
		Study documentation	Practical: • Contact logs and structured follow-up tracking • Audio-recorded sessions for fidelity	Maintained detailed documentation of encounters, follow-up attempts, and intervention fidelity including audio recordings of participant-CHW interactions
(Heisler et al. 2022) (Detroit CHW program)/CHW program vs. usual services	Traditional roles	Decreasing barriers	Didactic: • Core CHW competencies training (e.g., navigating social services, communication) • Training on needs assessment	Conducted social needs and behavioral health assessments; linked participants to housing, food, transportation, and behavioral health resources
		Health coaching	Practical: • Development of individualized action plans • Motivational techniques • Goal-setting strategies	Helped participants set goals and provided ongoing follow-up to support adherence to care plans and promote self-management
		Providing access to social services	Practical: • Resource mapping • Referral systems • Neighborhood-specific outreach strategies	Referred clients to community-based services and followed up to ensure successful connections
	Research roles	Study documentation	Practical: • Encounter logging and referral tracking • Shared progress updates in team meetings	Maintained contact logs, documented referrals, and engagement levels in structured data systems shared with health plans and evaluation teams
		Assisted adherence	Practical: • Patient follow-up protocols • Strategies to reduce ED use and encourage ambulatory care	Encouraged appropriate care use, educated about health system navigation, and supported reductions in ED utilization through ongoing coaching
(Ibe et al. 2019) (Triple P)/CHW coaching vs. minimal intervention	Traditional roles	Health coaching	Practical: • Structured patient-centered coaching protocols • Coaching on medication adherence and lifestyle modifications	Provided coaching on hypertension self-management, including adherence to medications, diet, physical activity, and stress management
		Educating	Didactic: • Educational materials (newsletter, photo-novel) • Hypertension education content	Reinforced hypertension knowledge using newsletters and scenario-based learning; explained disease-specific concerns during sessions
		Providing culturally appropriate services	Didactic: • CHWs indigenous to participants' communities • Shared linguistic and cultural background	Facilitated trust and communication through shared socio-cultural background and community linkage
	Research roles	Assisted adherence	Practical: • Monitoring coaching duration and topic tracking • Adherence discussion during structured follow-ups	Guided discussions on adherence; tracked number and type of coaching topics discussed (e.g., stress, lifestyle, medication)
		Study documentation	Practical: • Recording encounter length and topic frequency • Structured follow-up tracking	Logged each patient interaction and coaching topic to quantify CHW exposure
(Ibe et al. 2021) (RICH LIFE)/Collaborative care vs. usual care	Traditional roles	Decreasing barriers	Didactic: • Interprofessional training on health disparities and care team communication • Strategies for engagement	CHWs conducted SDOH assessments and connected patients to food, housing, and transportation support based on NCM referral
		Health coaching	Practical: • Joint planning with nurse care managers • Motivational techniques to encourage adherence	Used patient-centered communication to promote adherence to antihypertensive regimens and reinforce self-management
		Providing access to social services	Practical: • Community resource linkage protocol • Rapid referral workflow for urgent social needs	Linked patients with community resources for food insecurity, unstable housing, utilities, and domestic violence
	Research roles	Study documentation	Practical: • Documenting CHW referrals and encounters in care team logs • Use of structured protocols	Logged CHW activities, frequency of interaction, and type of social needs addressed; contributed to REDCap database and implementation evaluation
		Assisted adherence	Practical: • Shared protocol for identifying ongoing barriers • Outreach after missed NCM contact	Provided follow-up support for hard-to-reach patients; CHWs conducted home visits or met patients after clinical visits to support adherence and care continuity
(Islam et al. 2023) (IMPACT)/CHW-led coaching vs. single session (control)	Traditional roles	Educating	Didactic: • Adapted NHLBI Healthy Heart curriculum • Translated and culturally tailored materials • Group education facilitation	Delivered 5 monthly culturally tailored education sessions on HTN and self-management in primary care and community locations
		Health coaching	Practical: • Goal setting and behavior change support • Biweekly follow-ups via phone or in-person	Helped participants develop and follow action plans for BP management, medication adherence, PA, diet
		Providing culturally appropriate services	Didactic: • Language concordance • Community cultural context and family dynamics training	Delivered services in native languages (e.g., Bangla, Hindi); tailored examples to cultural/family practices
		Providing access to social services	Practical: • Navigation of food/mental health/community resources • Community partnership integration	Referred participants to local services (e.g., food pantries, mental health); connected via community-based organization network
	Research roles	Assisted adherence	Practical: • Medication adherence counseling • BP device training	Supported medication adherence via counseling; reinforced BP monitoring using Omron devices
		Study documentation	Practical: • Encounter tracking • Behavioral survey collection at 3 and 6 months	Documented BP, BMI, PROMIS, and self-reported behavior outcomes using structured tools and EHR supplements
Johansson et al. (2023) (RNCC/CHW Pilot)/RNCC/CHW team vs. standard primary care	Traditional roles	Educating	Didactic: • Education on CVD risk factors • Action planning and goal setting • Use of self-monitoring tools	Delivered individualized education using print booklets, reviewed self-monitoring logs, and used teach-back to reinforce CVD knowledge
		Health coaching	Practical: • Cognitive-behavioral goal setting • Self-monitoring with apps/logs • Problem-solving and feedback strategies	Held sessions focused on risk-specific goals (e.g., BP, cholesterol, physical activity); reinforced goals with feedback and confidence building
		Providing culturally appropriate services	Didactic: • Local adaptation of dietary/PA strategies • Cultural appropriateness for rural patients	Provided interventions tailored to rural access constraints (e.g., food access, fitness resources); emphasized practicality and community familiarity
	Research roles	Assisted adherence	Practical: • Reinforcement of medication and lifestyle goals • Coaching on app-based self-monitoring	Reinforced adherence to lifestyle changes and medication plans through CHW follow-up, self-monitoring logs, and supportive communication
		Study documentation	Practical: • CHW and RNCC field notes • Documentation of session counts, timing, and method (phone/in-person/video)	Logged intervention delivery via field notes; documented method, timing, and engagement with tools like apps and phone sessions
(Kangovi et al. 2017) (IMPaCT)/CHW intervention vs. usual care	Traditional roles	Decreasing barriers	Didactic: • CHW core competencies training (active listening, motivational interviewing) • Community resource navigation	Identified unmet health/social needs; removed structural barriers (transport, food, housing) that prevented goal achievement
		Health coaching	Practical: • Longitudinal goal-based coaching • Weekly 1-on-1 follow-ups	Supported personal goals through repeated coaching interactions in person and by phone
	Research roles	Study documentation	Practical: • Encounter logs • Fidelity checklists and supervision reports	Documented frequency and content of encounters and ensured intervention fidelity through supervisor monitoring
(Katula et al. 2017) (LIFT diabetes)/Lifestyle weight loss (CHW-led) vs. diabetes self-management (professional-led)	Traditional roles	Health coaching	Didactic: • Social cognitive theory and self-monitoring strategies • Weight management, goal setting, relapse prevention • Problem-solving, stimulus control, cognitive behavioral skills	CHWs facilitated weekly then monthly group sessions on behavioral weight loss, self-monitoring, and problem-solving using DVDs and toolkits; supported personalized health goals and action plans
		Educating	Didactic: • Diabetes and cardiovascular risk • Nutrition and caloric intake • Physical activity basics • Hypoglycemia prevention	Delivered structured education using adapted materials from Look AHEAD and HELP PD; reinforced healthy eating, physical activity, and glucose monitoring
		Providing culturally appropriate services	Didactic: • Recruited CHWs from the same community • Tailored education with cultural/linguistic relevance	CHWs conducted sessions in familiar community settings and built rapport using shared lived experience and community trust
	Research roles	Study documentation	Practical: • CHW tracking forms and web-based monitoring • Session attendance, weight, and adherence logs	CHWs tracked attendance, self-monitoring data, and weight; data was monitored by intervention team for fidelity and participant progress
		Assisted adherence	Practical: • Adherence coaching to diet and PA goals • Toolbox support for individual tailoring	Supported goal reinforcement during group weigh-ins and check-ins; tailored intervention tools to address barriers to adherence
(Krieger et al. 1999) (Seattle BP project)/CHW outreach vs. usual care	Traditional roles	Decreasing barriers	Practical: • Community resource training • Navigation of services like transportation, childcare, and clinic access	Assisted with access to appointments and support services including transportation and scheduling for follow-up BP care
		Providing culturally appropriate services	Didactic: • Community-based CHW recruitment • Shared cultural background training	CHWs were from same neighborhoods as participants and used culturally aligned communication approaches to build trust
		Educating	Didactic: • HTN and cardiovascular system • Risk factor education	Provided client education during measurement and follow-up contacts on HTN and the importance of follow-up
	Research roles	Study documentation	Practical: • Tracking follow-up attempts (calls, letters, home visits) • Computerized contact logs	Logged contact efforts (calls, letters, visits), maintained computerized records of appointment status and patient engagement
		Assisted adherence	Practical: • Appointment making and reminder protocols • Follow-up on missed visits	Made appointments, confirmed visits, sent reminders, and re-scheduled missed appointments to support clinical follow-up
(Levine et al. 2003) (project reducing BP)/CHW home education vs. control	Traditional roles	Educating	Didactic: • Six-session curriculum on CVD risk, medications, nutrition, and exercise	CHWs provided culturally tailored home-based educational sessions on BP, weight control, cholesterol, and physical activity
		Providing culturally appropriate services	Didactic: • Bilingual/bicultural CHW recruitment • Training on local cultural norms	Sessions delivered in participants' preferred language by CHWs from the same community
	Research roles	Study documentation	Practical: • Home visit documentation forms • Outcome tracking logs	Tracked completion of sessions and follow-up outcomes for each participant
(Margolius et al. 2012) (coaching & home titration)/Coaching + home titration vs. coaching only	Traditional roles	Health coaching	Practical: • 16–20 h of training on hypertension, medications, and lifestyle behavior change • Action plan development	CHWs conducted weekly phone/in-person sessions to support BP control, review logs, reinforce adherence, and adapt action plans
		Educating	Didactic: • Patient education on HTN goals and medications • Use of home BP monitor and lifestyle education tools	Educated patients on BP targets, medication understanding, diet, and exercise; provided training on BP logging using Omron devices
		Providing culturally appropriate services	Didactic: • Language-concordant training • Community-based hiring and communication styles	Coaches recruited from patient communities; sessions conducted in preferred language (English, Spanish, Cantonese, or Vietnamese)
	Research roles	Assisted adherence	Practical: • Medication adherence support • Action plan review and titration counseling	Reviewed adherence, offered support, and helped initiate med changes based on protocols after BP threshold triggers
		Contact the primary care team	Practical: • Coordination of med titration requests • Physician alerts and EHR entry	Communicated med changes with physicians, faxed prescriptions, and logged changes in EHR
		Study documentation	Practical: • Coaching encounter logs • BP tracking and titration activity documentation	Documented weekly BP readings, medication adherence, action plans, and changes; maintained detailed logs and fidelity tracking
(Samuel-Hodge et al. 2020) (CHANGE)/CHW coaching + lifestyle program vs. control	Traditional roles	Health coaching	Practical: • CVD risk management and motivational interviewing training • Group facilitation training	CHWs led group and individual coaching on healthy eating, physical activity, weight loss, and self-monitoring
		Educating	Didactic: • Eight-module culturally adapted manual on heart health	Provided community-based educational classes and follow-ups in churches and health centers
		Providing access to social services	Practical: • Referral pathways for lifestyle and clinical services	Referred participants to primary care and nutrition services
	Research roles	Study documentation	Practical: • Encounter logs • Participant progress tracking	Maintained logs for sessions attended, weight/BP progress, and program fidelity
(Shah et al. 2024) (DREAM Atlanta)/Telehealth CHW program vs. single education session (control)	Traditional roles	Educating	Didactic: • Curriculum on HTN, DMII, nutrition, physical activity, stress, and management • Culturally tailored dietary/exercise education	Delivered 5 culturally tailored virtual group sessions covering HTN and DMII topics, including culturally relevant food and exercise strategies
		Health coaching	Practical: • Action plan creation • Motivational interviewing techniques • Monthly follow-up coaching calls	CHWs conducted one-on-one follow-ups to review action plans and reinforce behavior change through motivational support
		Providing culturally appropriate services	Didactic: • CHWs from target community • Language and cultural congruence (e.g., Bengali/English)	Delivered education and coaching in participants' preferred language; aligned content with religious and cultural practices
	Research roles	Assisted adherence	Practical: • Home BP and weight monitoring education • Reinforcement of medication-taking behaviors	Supported self-monitoring using study-provided devices; reinforced adherence via follow-ups and action plan reviews
		Study documentation	Practical: • Progress notes • Call tracking and survey data collection	Logged participant contacts, session completion, and survey outcomes including BP, physical activity, and diet
(Stahl et al. 1977) (hypertension motivational interventions)/Community-based HT outreach vs. passive letter or gift techniques	Traditional roles	Educating	Didactic: • Basic hypertension knowledge • Communication skills • Outreach script and community trust building	Community members trained as HTs delivered health messages on BP risks and importance of screening during home visits
		Decreasing barriers	Practical: • Door-to-door outreach • Direct screening during visit • Flexible hours (weekends/evenings)	CHWs reduced access barriers by directly visiting homes to perform BP checks; no need for clinic attendance
		Being a member of the community	Didactic: • HTs recruited from same neighborhoods • Informal interpersonal approach	CHWs built trust and increased engagement due to shared background and community familiarity
	Research roles	Assisted adherence	Practical: • BP measurement training • Identification of high-risk individuals (≥95 diastolic)	Performed in-home blood pressure checks and identified undiagnosed HTN for further care
		Study documentation	Practical: • Household tracking • Categorization of intervention outcomes (BPM, refusals, known/unknown hypertension)	Recorded household responses, success of interventions, and percentage of new vs. known hypertensive cases identified

**Table 1B:** Summary of key studies with CHW roles in cardiovascular disease prevention.

**Study identifier/study design**	**CHW role type**	**Role definition**	**Training-specific to intervention**	**Delivered interventions**
(Dromerick et al. 2011) (PROTECT DC)/Community-based stroke navigator intervention vs. usual care	Traditional Roles	Addressing barriers	Didactic: • Motivational interviewing techniques • Practical problem-solving skills	Facilitated medication adherence; addressed transportation, insurance, and medication side-effect concerns
		Educating	Didactic: • Stroke awareness education • American Heart Association dietary guidelines • Physical activity education	Provided tailored stroke education and lifestyle counseling
	Research roles	Assisted adherence	Practical: • Medication adherence protocols • Pill counting procedures	Conducted home visits and monthly phone calls to ensure adherence
		Contact the primary care team	Practical: • Communication and liaison skills	Acted as liaison with primary care providers to manage patients' care
(Kitzman et al. 2017) (KC3T)/CHW navigation support vs. no structured transition support	Traditional roles	Educating	Didactic: • Stroke education and discharge preparation • Medication, mobility, caregiver training • Chronic disease management (HTN, DM, cholesterol, COPD) • Family/caregiver education	CHW (navigator) conducted in-home and phone follow-ups to reinforce stroke recovery education, chronic disease topics, and caregiver support over 6 months
		Health coaching	Practical: • Certified in CDSMP, DSMP, and WRAP • Goal setting and long-term transition coaching	Helped patients and families manage recovery, track rehab milestones, and stay engaged in stroke-specific goals; facilitated personalized care navigation
		Providing access to social services	Practical: • Resource navigation: DME, insurance, meds, home modifications • Health system coordination	Secured access to waiver programs, DME, and medical visits; served as advocate for access to care and continuity of services in rural low-resource areas
		Being a member of the community	Didactic: • Local CHW trained via Kentucky Homeplace • Culturally embedded outreach training	Navigator lived in and understood the local Appalachian community, building rapport and trust to improve service uptake and reduce health care distrust
	Research roles	Assisted adherence	Practical: • Medication compliance tracking • Appointment and rehab follow-up documentation	CHW monitored med adherence, provider appointments, rehab visits; used self-report and provider confirmation to track engagement
		Study documentation	Practical: • Encounter tracking system • KC3T custom database for risk factors and service uptake	Logged all education, contacts, resource access, compliance, and 30-day hospital readmission/ED visit outcomes using structured forms
(Towfighi et al. 2021) (SUCCEED)/CHW navigators + RN team vs. usual care	Traditional roles	Addressing barriers	Didactic: • Social needs screening and navigation training	CHWs performed social needs assessments and coordinated services for transportation, housing, and insurance
		Health coaching	Practical: • Stroke-specific self-management support • CHW monthly follow-up calls	Coached participants on medication adherence, lifestyle changes, and goal setting through home visits and calls
	Research roles	Study documentation	Practical: • Contact logs and team meeting notes • Fidelity tracking	Tracked CHW contacts, intervention delivery, and adherence; reviewed cases during clinical supervision
		Assisted adherence	Practical: • Reinforcement of secondary stroke prevention behaviors	Supported adherence to medications and appointments via regular education and follow-ups

COACH, counseling of adults for cardiovascular health; NP, nurse practitioner; CHW, community health worker; NHLBI, national heart, lung, and blood institute; BMI, body mass index; PROMIS, patient-reported outcomes measurement information system; EHR, electronic health record; HTN, hypertension; CVD, cardiovascular disease; BP, blood pressure; HBPM, home blood pressure monitoring; EMR, electronic medical record; A1c, glycated hemoglobin; ABCD, A1c, blood pressure, cholesterol, and depression; SUCCEED, stroke unmet needs and caregiver experiences evaluation and detail; AHH, ambulatory hypertension and health; ACT, achieve clinical targets; PCMH, patient-centered medical home; IMPACT, improving maintenance of patient-centered access to care and treatment; RNCC, registered nurse care coordinator; IMPaCT, individualized management for patient-centered tasks; LIFT, lifestyle intervention for tracking; CHANGE, community health action for neighborhood growth and empowerment; DREAM, diabetes reaching education and management; DMII, diabetes mellitus type II; HT, health technician; KC3T, Kentucky community care coordination by telehealth; SDOH, social determinants of health; NCM,; DM, diabetes mellitus; COPD, chronic obstructive pulmonary disease; ED, emergency department; CDSMP, chronic disease self-management program; DSMP, diabetes self-management program; WRAP, wellness recovery action plan; BPM, blood pressure monitoring; DME, durable medical equipment.

Regarding specific health behavior related interventions to improve outcomes, 12 studies discussed health behavior interactions, including nutrition (8 studies; Balcázar et al., 2010; Ephraim et al., 2014; Katula et al., 2017; Margolius et al., 2012; Shah et al., 2024; Towfighi et al., 2021; Islam et al., 2023; Samuel-Hodge et al., 2020), smoking cessation (4 studies; Becker et al., 2005; Ephraim et al., 2014; Katula et al., 2017; Margolius et al., 2012), lipid profile management (2 studies; Johansson et al., 2023; Katula et al., 2017), diabetes control (7 studies; Allen et al., 2014; Balcázar et al., 2010; Ephraim et al., 2014; Katula et al., 2017; Shah et al., 2024; Islam et al., 2023; Samuel-Hodge et al., 2020), hypertension management (10 studies; Allen et al., 2014; Balcazar et al., 2009; Balcázar et al., 2010; Ephraim et al., 2014; Katula et al., 2017; Krieger et al., 1999; Shah et al., 2024; Towfighi et al., 2021; Islam et al., 2023; Samuel-Hodge et al., 2020), and sleep (1 study; Towfighi et al., 2021), with limited detail on these interactions focusing on CHW promotion of these behaviors. Furthermore, 15 studies encouraged PA, using methods like personalized exercise plans (Allen et al., 2014; Ephraim et al., 2014; Katula et al., 2017; Shah et al., 2024; Towfighi et al., 2021; Islam et al., 2023; Samuel-Hodge et al., 2020), group activities (Balcázar et al., 2010; Ephraim et al., 2014; Katula et al., 2017; Shah et al., 2024; Samuel-Hodge et al., 2020), and motivational interviewing (Allen et al., 2014; Becker et al., 2005; Ephraim et al., 2014; Katula et al., 2017; Krieger et al., 1999; Margolius et al., 2012; Shah et al., 2024; Towfighi et al., 2021; Islam et al., 2023; Samuel-Hodge et al., 2020); 13 studies (Allen et al., 2014; Balcazar et al., 2009; Balcázar et al., 2010; Ephraim et al., 2014; Johansson et al., 2023; Katula et al., 2017; Krieger et al., 1999; Margolius et al., 2012; Shah et al., 2024; Towfighi et al., 2021; Islam et al., 2023; Samuel-Hodge et al., 2020; Dromerick et al., 2011) included PA as a preventive component; and 10 studies (Allen et al., 2014; Balcazar et al., 2009; Balcázar et al., 2010; Ephraim et al., 2014; Katula et al., 2017; Margolius et al., 2012; Shah et al., 2024; Towfighi et al., 2021; Islam et al., 2023; Samuel-Hodge et al., 2020) integrated PA into broader lifestyle interventions aimed at hypertension control, weight management, or diabetes prevention. These cumulative interventions typically included PA alongside diet, medication adherence, and self-monitoring components. While most studies addressed PA through tracking tools or behavioral coaching, none focused solely on PA as the primary intervention target. Instead, PA was often embedded within multicomponent behavioral programs.

Among the three trials for secondary stroke prevention (Towfighi et al., 2021; Dromerick et al., 2011; Kitzman et al., 2017), both PROTECT DC (Dromerick et al., 2011) and the Stroke Unmet Needs and Caregiver Experiences Evaluation and Detail (SUCCEED; Towfighi et al., 2021) tested self-management interventions that included coaching, education, and social support. PROTECT DC was only reported as a feasibility study protocol. SUCCEED reported improvements in self-management and medication adherence. Using PA as an intervention, SUCCEED (Towfighi et al., 2021) reported that 50%−65% of participants engaged in weekly exercise (mean: 30–60 min), while PROTECT DC (Dromerick et al., 2011) promoted PA through coaching but did not quantify engagement. SUCCEED also targeted hypertension control, sodium reduction, and successfully addressed social needs, including assistance with transportation and housing instability, food insecurity, and medication cost. The Kentucky Community Care Coordination by Telehealth (KC3T) study (Kitzman et al., 2017) focused on post-stroke recovery. The researchers found improvements in patient self-management and access to supportive services and fewer hospital readmissions.

### CHW roles and responsibilities in interventions

Across the 24 studies reviewed, CHWs played an integral role in delivering interventions, both through traditional responsibilities and research-related tasks. In 17 studies, CHWs acted as the sole intervention deliverer. In the remaining seven (Becker et al., 2005; Heisler et al., 2022; Ibe et al., 2021; Johansson et al., 2023; Katula et al., 2017; Towfighi et al., 2021; Kitzman et al., 2017), they collaborated with nurse practitioners, social workers, pharmacists, or other professionals, highlighting the adaptable nature of CHWs within various multidisciplinary care teams. Overall, CHWs frequently delivered culturally tailored interventions (21 studies), enhanced patient engagement (19 studies), and addressed barriers to care (15 studies), leveraging community ties to improve access and trust. All 24 studies utilized CHWs in traditional roles, with education (23 studies) and health coaching (22 studies) being the most frequent, followed by providing culturally appropriate services (18 studies). Participation in research roles occurred in 21 studies, including active participation to improve compliance with trial procedures (20 studies), data collection (19 studies), and communication with the study's primary care team (9 studies). In the three stroke studies, traditional CHW roles included education, coaching, and cultural tailoring, and research roles encompassed adherence support and documentation, while two included care coordination.

### Training provided to CHWs for intervention delivery

The standard training provided to CHWs included disease-specific education (22 studies), motivational interviewing or related behavioral coaching and cultural sensitivity training (15 studies; Balcazar et al., 2009; Balcázar et al., 2010; Becker et al., 2005; Commodore-Mensah et al., 2024; Ephraim et al., 2014; Heisler et al., 2022; Katula et al., 2017; Margolius et al., 2012; Shah et al., 2024; Towfighi et al., 2021; Samuel-Hodge et al., 2020; Kitzman et al., 2017; Ell et al., 2017), For research roles, frequent intervention-specific trainings were on protocol adherence (Allen et al., 2014; Balcazar et al., 2009; Balcázar et al., 2010; Becker et al., 2005; Commodore-Mensah et al., 2024; Ephraim et al., 2014; Katula et al., 2017; Margolius et al., 2012; Shah et al., 2024; Towfighi et al., 2021; Kitzman et al., 2017; Ell et al., 2017), data collection methods (Allen et al., 2014; Balcázar et al., 2010; Becker et al., 2005; Commodore-Mensah et al., 2024; Ephraim et al., 2014; Katula et al., 2017; Margolius et al., 2012; Shah et al., 2024; Towfighi et al., 2021; Islam et al., 2023; Ell et al., 2017), and data gathering with blood pressure (BP) monitoring techniques (Allen et al., 2014; Commodore-Mensah et al., 2024; Katula et al., 2017; Margolius et al., 2012; Shah et al., 2024; Towfighi et al., 2021; Islam et al., 2023; Ell et al., 2017), reflecting CHWs' critical contributions to study execution. Of these studies, the individualized management for patient-centered tasks (IMPaCT) trial (Kangovi et al., 2017) had the most comprehensive and replicable training model, with detailed online material posted for the highest transparency and fidelity. The challenges to training include inconsistent curricula, limited funding, and unclear roles, while success depends on factors such as community trust, well-defined responsibilities, and institutional backing (O'Brien et al., 2009; Mallaiah et al., 2023; see Table 1 and the Supplemental material).

In non-stroke studies, such as the LINKED-HEARTS program (Commodore-Mensah et al., 2024), CHWs trained in home BP monitoring, telemonitoring via the Sphygmo application, and electronic medical record documentation. They were also trained to deliver telehealth follow-ups and ensure accurate BP data transmission. In the Counseling of Adults for Cardiovascular Health trial (Allen et al., 2014), CHWs were trained to provide tailored education and goal setting. In the CHANGE study (Samuel-Hodge et al., 2020), CHWs were integrated within faith-based networks to promote sustainable dietary and lifestyle modifications.

All three stroke studies provided disease-specific education, motivational interviewing, and protocol adherence training to the CHWs. SUCCEED and KC3T (Towfighi et al., 2021; Kitzman et al., 2017) clearly identified the CHWs' intervention delivery by specifying the training duration, balancing didactic and practical components in the intervention group. SUCCEED trained CHWs in social needs screening and stroke self-management, with monthly coaching to address barriers such as transportation, housing, or medication access. KC3T trained CHWs in stroke education and resource navigation, facilitating in-home follow-ups and access to durable medical equipment, thereby enhancing stroke recovery. CHW training across these studies included practical tools and communication strategies that ensured intervention fidelity, with telehealth (SUCCEED), in-home monitoring (KC3T), nurse collaboration, and resource linkage demonstrating CHWs' flexibility in varied care delivery models.

### CHW integration in study teams

CHWs' integration into multidisciplinary teams was noted in 10 studies (Allen et al., 2014; Ephraim et al., 2014; Heisler et al., 2022; Ibe et al., 2021; Johansson et al., 2023; Katula et al., 2017; Shah et al., 2024; Towfighi et al., 2021; Islam et al., 2023; Kitzman et al., 2017). The methods used to engage CHWs in research procedures included regular team meetings (Allen et al., 2014; Ephraim et al., 2014; Katula et al., 2017; Shah et al., 2024; Towfighi et al., 2021), shared decision-making with care providers (e.g., nurses, pharmacists, physicians, Katula et al., 2017; Shah et al., 2024; Towfighi et al., 2021), and clear role definitions, including scope of practice for tasks like BP telemonitoring (Allen et al., 2014; Ephraim et al., 2014; Ibe et al., 2021; Katula et al., 2017; Shah et al., 2024; Towfighi et al., 2021).

Three studies (Heisler et al., 2022; Islam et al., 2023; Kitzman et al., 2017) reported the integration of CHWs into their academic institutions. Five studies reported barriers like institutional review board (IRB) challenges (Heisler et al., 2022; Towfighi et al., 2021; Kitzman et al., 2017) and salary support (Heisler et al., 2022; Islam et al., 2023). To facilitate research roles, only five studies (Ephraim et al., 2014; Heisler et al., 2022; Islam et al., 2023; Kitzman et al., 2017; Towfighi et al., 2017) trained CHWs in consent processes, with IRB-directed training for the conduct of study procedures. Among the three stroke studies, two (Towfighi et al., 2021; Kitzman et al., 2017) reported CHW IRB training, with one (Towfighi et al., 2021) involving participant consent. Institutional facilitators included team meetings (Katula et al., 2017; Shah et al., 2024; Towfighi et al., 2017) and role clarity (Margolius et al., 2012; Samuel-Hodge et al., 2020; Kitzman et al., 2017), while IRB delays were a barrier for one study (Kitzman et al., 2017).

Based on our review of co-authors' lists in the 24 trial results publications, CHWs were listed as co-authors in two of the included studies (Balcazar et al., 2009; Kitzman et al., 2017). Four studies included them in the acknowledgment section (Heisler et al., 2022; Islam et al., 2023; Kitzman et al., 2017; Ibe et al., 2019).

## Discussion

We reviewed 24 studies that targeted CHW-engaged cardiovascular health promotion to prevent CVD in the United States. We found that all studies included some, if not all, components of the AHA Life's Essential 8 health behavior change recommendations (Lloyd-Jones et al., 2022). CHWs served alongside other disciplines in traditional roles to conduct these lifestyle interventions, of which PA and hypertension control were most emphasized. All CHWs were trained in disease-specific education, and in most studies (17 of 24), they were trained for effective communication and cultural competency, but didactics and practical training methods varied. While most studies (23 of 24) engaged CHWs in essential research roles, such as intervention adherence, study documentation, and team coordination, only half documented research training, and only four acknowledged one or more CHWs in study publications. Only 3 of the 24 papers specifically engaged stroke populations.

We found that CHWs perform a crucial role in health services outreach across the spectrum of community-based research (Key et al., 2019). Furthermore, they can catalyze behavioral modifications for vascular risk factors that are widely applicable to CVD prevention for multiple chronic diseases. Evidence from systematic reviews shows that CHWs can lower systolic blood pressure by 5–10 mmHg and boost medication adherence by up to 20% in underserved groups (Brownstein et al., 2007; Jacob et al., 2019). CHWs have been engaged in effectively promoting health behaviors, advocating for health access, and aiding adherence to treatments for chronic conditions like hypertension and diabetes, both major heart disease and stroke risk factors since the 1960s (Viswanathan et al., 2010; Perry et al., 2014). While most studies included CHWs engaged behavioral modifications to target these conditions, only three studies utilized CHWs specifically for secondary stroke prevention (Towfighi et al., 2021; Dromerick et al., 2011; Kitzman et al., 2017).

### Gaps and opportunities

CHW training to conduct CVD interventions is essential but varies widely. Intervention-specific training, combining theoretical knowledge (e.g., disease mechanisms and behavioral theories) with practical skills (e.g., blood pressure monitoring and data recording), varied considerably (O'Brien et al., 2009; Allen et al., 2015). Differences in training rigor suggest that thoughtful transfer of knowledge and skills training for CHWs is needed to extend their skill set beyond traditional roles or fundamental research roles tailored to each intervention. It also highlights a need for rigorous assessment tools to evaluate CHWs' knowledge and skills to optimize their performance and enhance their credibility in CVD interventions, as previously noted by (Mallaiah et al. 2023).

From our included studies, it is apparent that CHWs were incorporated into multidisciplinary intervention teams. Within multidisciplinary teams, CHWs have been previously found to improve patient engagement, as demonstrated in initiatives targeting stroke risk factors (Towfighi et al., 2021; Kitzman et al., 2017). Harnessing their expertise to bridge health care delivery and provide social support at a time when health care is most fragmented, such as transitions of care from the hospital into the community (Reeves et al., 2023), may be particularly beneficial and cost-effective. However, working collaboratively in teams should be reflected in their training. For example, for future secondary stroke prevention interventions, CHW training would need to include didactics and practical training for physical and cognitive disabilities among stroke patients. As the length of stay in the hospital (Bettger et al., 2019) and first-year post-stroke rehabilitation services are low in the United States (Young et al., 2023), patients are more vulnerable to ineffective community reintegration and poor recovery. These gaps can be bridged by CHWs, who should engage with other health care professionals, such as rehabilitation clinicians, to provide training for safe and effective transfer of knowledge and skills. CHWs could then be leveraged in stroke trials to develop stroke-specific competencies in stroke disability accommodations and care transitions from facilities to home for safety assessments and rehabilitation services.

In the United States, CHW integration can be expanded to improve care among minoritized populations. While CHWs have been prominently engaged in LMICs with a focus on eliminating health care disparities, CHW interventions have now emerged as a promising approach among underserved settings in the United States (Spencer et al., 2010). Their role in low-resource and income settings can be instrumental to creating health care equity for community resource building. They can lift the community they serve and expand their impact if equipped with leadership roles by academic partnerships in community-based participatory research (CBPR). For example, in the REACH Detroit Partnership Family Intervention, CHWs played a major role in the development and implementation of the project's culturally tailored Journey to Health/Camino a la Salud diabetes education curriculum (Feathers et al., 2007).

Furthermore, our scoping review shows that while CHWs can be engaged in traditional roles to support communities, research-supported roles that are primarily acquired through institution-based training in an academic center need to be improved. While all interventions provided some training for these roles, these were not clearly reported. Transparency in academic center training for CHWs to engage with the community would be helpful to standardize this approach across institutions. Providing CHW training beyond preventive care to skills so that they can serve as paraprofessionals within defined interventions and extending licensed health care professionals can serve to propel health services research after a cardiovascular or cerebrovascular event. This would also help create a bidirectional capacity-building framework for community-based health workers and academic institutions, expanding the role of community health workers in an academic institution as well as providing further support in the community. The training framework suggested by the U.S. Agency of International Development for the CHW Assessment and Improvement Matrix provides a clear toolkit for implementing and strengthening CHW programs and services, allowing them to serve as key health care workers in underserved areas (Crigler et al., 2013). Furthermore, established frameworks in implementation science, such as the Consolidated Framework for Implementation Research, can be utilized to evaluate intervention delivery by CHWs, specifically in the context of training, fidelity, and sustainability (Damschroder et al., 2022). Thus, by expanding their roles in a standardized framework, facilitated by the academic institution, CHWs can have a central role in culturally congruent interventions across the CBPR spectrum.

CHW interventions can serve as a low-cost investment to provide academic institutions with the incentive and scope to develop interventions that best serve underserved populations to improve health care utilization and therefore provide equitable care. Having trained CHWs utilize billing codes under Medicaid, together with research funding for preventive interventions, may make clinical care more cost-effective and sustainable to maintain (HealthySteps National Office Policy Finance Team, 2024). However, the eligibility of CHW services for Medicaid billing varies by state, and reimbursement is nuanced by the services delivered and the burden of documentation. Therefore, by intentionally expanding the engagement of CHWs with academic institutions, bidirectional capacity building can be encouraged to lift community members who can serve their communities with longitudinal and long-term support from academic institutions that may be well-placed in these communities to serve them.

### Study limitations

Our review has several limitations. First, it is limited by challenges related to utilizing one database with a search methodology using keywords, which can be inconsistent in terminology across the literature. However, we used several broad keywords and reviewed the reference literature to ensure that our search was as robust as possible. Second, we only included articles published in English, possibly excluding studies not published with an English translation. Because our scope of interest was to include studies in the United States, where the primary language is English, we hope that we were able to capture all studies involving U.S. settings. Furthermore, studies serving non-English-speaking U.S. populations were still included as they were published in English. Third, publication bias is very possible as smaller trials with null results may have been less likely to have been published. Finally, we did not include literature on CHW perspectives on the interventions conducted, as it was outside the scope of this review, but we have included CHWs as co-authors of this review for their opinions.

## Conclusion

In summary, CHWs play an important role in bridging health care delivery to improve cardiovascular health. Growing CHW competencies and integration in multidisciplinary teams has the potential to address gaps in secondary stroke prevention trials, forging a pathway for robust academic institution–community partnerships and equitable care. As tangible next steps, developing CHW-led interventions that utilize standardized frameworks for CHW training may improve intervention fidelity, and establishing norms for CHW co-authorship may promote equity among health care workforces.
