# Supplementary material for: Community health worker roles in intervention delivery: a scoping review of heart disease and stroke prevention trials in the United States
Source: Front Stroke. 2025 Oct 2;4:1658612. doi: 10.3389/fstro.2025.1658612 (PMC12802658; doi:10.3389/fstro.2025.1658612)
Supplement: Supplementary file 1 [file Supplementary_file_1.docx]

**SUPPL_1_Summary of All Studies:**

**Cardiovascular Studies (21 studies)**

1. **Allen et al. 2014 (COACH) / Allen et al. 2011 (Protocol for COACH)**
   - **Target Population and Disease**: Adults with hypertension (cardiovascular).
   - **Nature of Intervention**: Primary prevention (hypertension management).
   - **Health-Behavior Interactions**: Hypertension management, medication adherence, lifestyle modifications (diet, physical activity).
   - **Measures Used**: Lifestyle counseling, drug adherence counseling, behavioral goal setting.
   - **Integration of CHWs**: CHWs reinforced nurse practitioner (NP) instructions, developed behavior change plans, and provided individualized adherence support using motivational interviewing.
   - **Barriers/Facilitators**: Not explicitly mentioned.
2. **Balcázar et al. 2009 (Promotoras de Salud Contra la Hipertension)**
   - **Target Population and Disease**: Hispanic adults with hypertension (cardiovascular).
   - **Nature of Intervention**: Primary prevention (hypertension management).
   - **Health-Behavior Interactions**: Hypertension management, nutrition, physical activity.
   - **Measures Used**: Educational modules, photonovela, family-centered coaching.
   - **Integration of CHWs**: CHWs (promotoras) delivered weekly sessions, guided participants through culturally tailored vignettes, and facilitated family involvement.
   - **Barriers/Facilitators**: Not explicitly mentioned; implicit facilitator: bilingual CHWs from the community likely enhanced trust.
3. **Balcázar et al. 2010 (HEART)**
   - **Target Population and Disease**: Adults at risk for cardiovascular disease (cardiovascular).
   - **Nature of Intervention**: Primary prevention (cardiovascular risk reduction).
   - **Health-Behavior Interactions**: Hypertension management, nutrition, physical activity, stress management.
   - **Measures Used**: Educational modules, goal setting, action planning.
   - **Integration of CHWs**: CHWs delivered group sessions, encouraged action steps, and provided follow-up support.
   - **Barriers/Facilitators**: Not explicitly mentioned.
4. **Becker et al. 2005 (Community-Based Care)**
   - **Target Population and Disease**: Adults with coronary heart disease risk factors (cardiovascular).
   - **Nature of Intervention**: Primary prevention (CHD risk reduction).
   - **Health-Behavior Interactions**: Hypertension management, lifestyle modifications (diet, physical activity, smoking cessation), medication adherence.
   - **Measures Used**: Counseling, action planning, self-monitoring.
   - **Integration of CHWs**: CHWs provided counseling, facilitated structured follow-ups, and connected participants to resources like YMCA memberships.
   - **Barriers/Facilitators**: Not explicitly mentioned.
5. **Commodore-Mensah et al. 2024 (LINKED-HEARTS)**
   - **Target Population and Disease**: Adults with hypertension and/or diabetes (cardiovascular).
   - **Nature of Intervention**: Primary prevention (hypertension and diabetes management).
   - **Health-Behavior Interactions**: Hypertension management, diabetes control, lifestyle modifications.
   - **Measures Used**: Culturally relevant lifestyle counseling, individualized care plans, biweekly coaching.
   - **Integration of CHWs**: CHWs provided education at patient-preferred locations, developed care plans, and linked participants to community services.
   - **Barriers/Facilitators**: Not explicitly mentioned.
6. **Daniels et al. 2012 (ABCD)**
   - **Target Population and Disease**: African American adults with cardiovascular risk factors (cardiovascular).
   - **Nature of Intervention**: Primary prevention (cardiovascular risk reduction).
   - **Health-Behavior Interactions**: Hypertension management, diabetes control, cholesterol management, depression.
   - **Measures Used**: Interactive education, return demonstration, peer modeling.
   - **Integration of CHWs**: CHWs delivered 6-week sessions, modeled positive behaviors, and provided peer counseling between sessions.
   - **Barriers/Facilitators**: Implicit facilitator: CHWs recruited from churches, leveraging community trust.
7. **Ell et al. 2017 (AHH) / Ell et al. 2016 (Protocol for AHH)**
   - **Target Population and Disease**: Adults with diabetes and/or heart disease (cardiovascular).
   - **Nature of Intervention**: Primary and secondary prevention (depression and chronic illness management).
   - **Health-Behavior Interactions**: Depression management, self-care for chronic diseases (diabetes, heart disease), social needs.
   - **Measures Used**: Problem-solving framework, goal setting, resource navigation.
   - **Integration of CHWs**: Community Health Workers (CHWs) supported behavior change through action plans, connected participants to social services, and delivered culturally tailored interventions.
   - **Barriers/Facilitators**: Not explicitly mentioned.
8. **Ephraim et al. 2014 (ACT)**
   - **Target Population and Disease**: Adults with hypertension (cardiovascular).
   - **Nature of Intervention**: Primary prevention (hypertension management).
   - **Health-Behavior Interactions**: Hypertension management, lifestyle modifications.
   - **Measures Used**: National Heart, Lung, and Blood Institute (NHLBI) “With Every Heartbeat is Life” manual, goal setting, action planning.
   - **Integration of CHWs**: CHWs provided culturally tailored education, supported goal setting, and linked patients to clinical and community resources.
   - **Barriers/Facilitators**: Not explicitly mentioned.
9. **Heisler et al. 2022 (Detroit CHW Program)**
   - **Target Population and Disease**: Adults with chronic diseases (cardiovascular focus).
   - **Nature of Intervention**: Primary prevention (chronic disease management).
   - **Health-Behavior Interactions**: Chronic disease management, social determinants of health (SDOH).
   - **Measures Used**: Needs assessment, goal setting, resource navigation.
   - **Integration of CHWs**: CHWs conducted assessments, developed action plans, and provided follow-up support.
   - **Barriers/Facilitators**: Not explicitly mentioned.
10. **Ibe et al. 2019 (Triple P)**
    - **Target Population and Disease**: Adults with hypertension (cardiovascular).
    - **Nature of Intervention**: Primary prevention (hypertension self-management).
    - **Health-Behavior Interactions**: Hypertension management.
    - **Measures Used**: Coaching on medication adherence, lifestyle modifications, motivational interviewing.
    - **Integration of CHWs**: CHWs provided coaching, reinforced hypertension knowledge, and supported adherence.
    - **Barriers/Facilitators**: Not explicitly mentioned.
11. **Ibe et al. 2021 (RICH LIFE)**
    - **Target Population and Disease**: Adults with hypertension (cardiovascular).
    - **Nature of Intervention**: Primary prevention (hypertension management).
    - **Health-Behavior Interactions**: Hypertension management, social determinants of health (SDOH).
    - **Measures Used**: Hypertension management, social determinants of health (SDOH).
    - **Integration of CHWs**: CHWs conducted SDOH assessments, connected patients to resources, and supported adherence through patient-centered communication.
    - **Barriers/Facilitators**: Not explicitly mentioned.
12. **Islam et al. 2023 (IMPACT)**
    - **Target Population and Disease**: Adults with hypertension (cardiovascular).
    - **Nature of Intervention**: Primary prevention (hypertension management).
    - **Health-Behavior Interactions**: Hypertension management, lifestyle modifications (diet, physical activity).
    - **Measures Used**: Culturally tailored education, goal setting, biweekly follow-ups.
    - **Integration of CHWs**: CHWs delivered education in primary care and community settings, supported action plans, and provided follow-up coaching.
    - **Barriers/Facilitators**: Not explicitly mentioned.
13. **Johansson et al. 2023 (RNCC/CHW Pilot)**
    - **Target Population and Disease**: Adults with cardiovascular risk factors (cardiovascular).
    - **Nature of Intervention**: Primary prevention (cardiovascular risk reduction).
    - **Health-Behavior Interactions**: Hypertension management, cholesterol management, physical activity.
    - **Measures Used**: Education on CVD risk factors, action planning, self-monitoring tools (apps, logs).
    - **Integration of CHWs**: CHWs delivered individualized education, reviewed self-monitoring logs, and used teach-back to reinforce knowledge.
    - **Barriers/Facilitators**: Not explicitly mentioned.
14. **Kangovi et al. 2017 (IMPaCT)**
    - **Target Population and Disease**: Adults with chronic diseases (cardiovascular focus).
    - **Nature of Intervention**: Primary prevention (chronic disease management).
    - **Health-Behavior Interactions**: Chronic disease management, social determinants of health (SDOH).
    - **Measures Used**: Goal setting, coaching, resource navigation.
    - **Integration of CHWs**: CHWs supported personal health goals, provided weekly coaching, and linked patients to community resources.
    - **Barriers/Facilitators**: Not explicitly mentioned.
15. **Katula et al. 2017 (LIFT Diabetes)**
    - **Target Population and Disease**: Adults with diabetes and cardiovascular risk factors (cardiovascular).
    - **Nature of Intervention**: Primary prevention (diabetes and cardiovascular risk management).
    - **Health-Behavior Interactions**: Diabetes control, hypertension management, weight loss.
    - **Measures Used**: Group sessions on weight management, self-monitoring, problem-solving.
    - **Integration of CHWs**: CHWs facilitated group sessions, supported self-monitoring, and tailored interventions using DVDs and toolkits.
    - **Barriers/Facilitators**: Not explicitly mentioned.
16. **Krieger et al. 1999 (Seattle BP Project)**
    - **Target Population and Disease**: Adults with hypertension (cardiovascular).
    - **Nature of Intervention**: Primary prevention (hypertension management).
    - **Health-Behavior Interactions**: Hypertension management.
    - **Measures Used**: Education on hypertension, appointment reminders, follow-up on missed visits.
    - **Integration of CHWs**: CHWs provided education during BP measurements, made appointments, and sent reminders.
    - **Barriers/Facilitators**: Not explicitly mentioned.
17. **Levine et al. 2003 (Project Reducing BP)**
    - **Target Population and Disease**: Adults with hypertension (cardiovascular).
    - **Nature of Intervention**: Primary prevention (hypertension management).
    - **Health-Behavior Interactions**: Hypertension management.
    - **Measures Used**: Home-based education on BP, weight control, and physical activity.
    - **Integration of CHWs**: CHWs delivered culturally tailored education in participants’ homes.
    - **Barriers/Facilitators**: Not explicitly mentioned.
18. **Margolius et al. 2012 (Coaching & Home Titration)**
    - **Target Population and Disease**: Adults with hypertension (cardiovascular).
    - **Nature of Intervention**: Primary prevention (hypertension management).
    - **Health-Behavior Interactions**: Hypertension management.
    - **Measures Used**: Coaching, home BP monitoring, medication titration support.
    - **Integration of CHWs**: CHWs conducted weekly coaching, supported BP monitoring, and facilitated medication changes.
    - **Barriers/Facilitators**: Not explicitly mentioned.
19. **Samuel-Hodge et al. 2020 (CHANGE)**
    - **Target Population and Disease**: Adults with cardiovascular risk factors (cardiovascular).
    - **Nature of Intervention**: Primary prevention (cardiovascular risk reduction).
    - **Health-Behavior Interactions**: Hypertension management, lifestyle modifications (diet, physical activity, weight loss).
    - **Measures Used**: Coaching, education, lifestyle program.
    - **Integration of CHWs**: CHWs led group and individual coaching on heart health and self-monitoring.
    - **Barriers/Facilitators**: Not explicitly mentioned.
20. **Shah et al. 2024 (DREAM Atlanta)**
    - **Target Population and Disease**: Adults with hypertension and/or diabetes (cardiovascular).
    - **Nature of Intervention**: Primary prevention (hypertension and diabetes management).
    - **Health-Behavior Interactions**: Hypertension management, diabetes control, lifestyle modifications (nutrition, physical activity, stress).
    - **Measures Used**: Virtual education sessions, action plan creation, motivational interviewing.
    - **Integration of CHWs**: CHWs delivered culturally tailored virtual group sessions and provided one-on-one follow-up coaching.
    - **Barriers/Facilitators**: Not explicitly mentioned.
21. **Stahl et al. 1977 (Hypertension Motivational Interventions)**
    - **Target Population and Disease**: Adults with hypertension (cardiovascular).
    - **Nature of Intervention**: Primary prevention (hypertension management).
    - **Health-Behavior Interactions**: Hypertension management.
    - **Measures Used**: Door-to-door outreach, education, BP screening.
    - **Integration of CHWs**: CHWs (Health Technicians) delivered health messages and performed BP checks at home.
    - **Barriers/Facilitators**: Not explicitly mentioned.

**Cerebrovascular Studies (3 studies)**

1. **Dromerick et al. 2011 (PROTECT DC)**
   - **Target Population and Disease**: Stroke survivors (cerebrovascular).
   - **Nature of Intervention**: Secondary prevention and recovery.
   - **Health-Behavior Interactions**: Medication adherence, lifestyle modifications (diet, physical activity).
   - **Measures Used**: Motivational interviewing, stroke education, problem-solving for barriers.
   - **Integration of CHWs**: CHWs facilitated medication adherence, provided tailored education, and addressed barriers like transportation and insurance.
   - **Barriers/Facilitators for CHWs**: Not explicitly mentioned.
2. **Kitzman et al. 2017 (KC3T)**
   - **Target Population and Disease**: Stroke survivors in rural areas (cerebrovascular).
   - **Nature of Intervention**: Recovery.
   - **Health-Behavior Interactions**: Chronic disease management (hypertension, diabetes), rehabilitation, caregiver support.
   - **Measures Used**: Stroke education, goal setting, resource navigation (e.g., durable medical equipment).
   - **Integration of CHWs**: CHWs conducted in-home and phone follow-ups, provided education, and facilitated access to rehabilitation and clinical resources.
   - **Barriers/Facilitators for CHWs**: CHWs trained via Kentucky Homeplace and recruited locally, enhancing trust and cultural alignment.
3. **Towfighi et al. 2021 (SUCCEED)**
   - **Target Population and Disease**: Stroke survivors (cerebrovascular).
   - **Nature of Intervention**: Secondary prevention and recovery.
   - **Health-Behavior Interactions**: Medication adherence, lifestyle modifications, social needs (e.g., housing, transportation).
   - **Measures Used**: Monthly coaching, social needs screening, self-management support.
   - **Integration of CHWs**: CHWs delivered coaching, screened for social needs, and supported stroke self-management.
   - **Barriers/Facilitators**: Not explicitly mentioned.

**SUPPL_2_MeSH terms**

("Community Health Workers"[Mesh] OR "community health representative" OR "community outreach worker" OR "community educator" OR "community care coordinator" OR "community health aide")

("Stroke"[Mesh] OR "Ischemic Stroke"[Mesh] OR "Stroke Rehabilitation"[Mesh] OR "Stroke, Lacunar"[Mesh] OR "cerebrovascular incident")

("Hyperlipidemias[Mesh]" OR "HLD")

("Hypertension"[Mesh] OR "HTN")

("Coronary Artery Disease"[Mesh] OR "CAD")

("Cardiovascular Diseases"[Mesh] OR "CVD")

("Myocardial Infarction"[Mesh] OR "Inferior Wall Myocardial Infarction"[Mesh] OR "Anterior Wall Myocardial Infarction"[Mesh] OR "Non-ST Elevated Myocardial Infarction"[Mesh] OR "ST Elevation Myocardial Infarction"[Mesh] OR "MI")

("home based exercise" OR "home-based exercise" OR "Exercise Therapy"[Mesh] OR "physical therapy" OR "physiotherapy")

("Heart Arrest"[Mesh] OR "Heart Arrest, Induced"[Mesh] OR "Out-of-Hospital Cardiac Arrest"[Mesh])

"Heart Failure"[Mesh] OR "CHF"

AND ((clinicaltrial[Filter] OR meta-analysis[Filter] OR randomizedcontrolledtrial[Filter] OR review[Filter] OR systematicreview[Filter]) AND (english[Filter]))

"Community Health Workers"[Mesh] and "Exercise Therapy"[Mesh]

"Community Health Workers"[Mesh] and "Physical Therapy"

"Community Health Workers"[Mesh] and "Stroke"[Mesh]

"Community Health" and "Stroke"[Mesh] and "Exercise"

Parameters

("Community Health Workers"[Mesh] OR "community health representative" OR "community outreach worker" OR "community educator" OR "community care coordinator" OR "community health aide") AND ("Coronary Artery Disease"[Mesh] OR "CAD") AND ((clinicaltrial[Filter] OR meta-analysis[Filter] OR randomizedcontrolledtrial[Filter] OR review[Filter] OR systematicreview[Filter]) AND (english[Filter]))

(("Community Health Workers"[MeSH Terms] OR "community health representative"[All Fields] OR "community outreach worker"[All Fields] OR "community educator"[All Fields] OR "community care coordinator"[All Fields] OR "community health aide"[All Fields]) AND ("Cardiovascular Diseases"[MeSH Terms] OR "CVD"[All Fields])) AND ((clinicaltrial[Filter] OR meta-analysis[Filter] OR randomizedcontrolledtrial[Filter] OR review[Filter] OR systematicreview[Filter]) AND (english[Filter]))

("Community Health Workers"[Mesh] OR "community health representative" OR "community outreach worker" OR "community educator" OR "community care coordinator" OR "community health aide") AND ("Myocardial Infarction"[Mesh] OR "Inferior Wall Myocardial Infarction"[Mesh] OR "Anterior Wall Myocardial Infarction"[Mesh] OR "Non-ST Elevated Myocardial Infarction"[Mesh] OR "ST Elevation Myocardial Infarction"[Mesh] OR "MI") AND ((clinicaltrial[Filter] OR meta-analysis[Filter] OR randomizedcontrolledtrial[Filter] OR review[Filter] OR systematicreview[Filter]) AND (english[Filter]))

("Community Health Workers"[Mesh] OR "community health representative" OR "community outreach worker" OR "community educator" OR "community care coordinator" OR "community health aide") AND ("Heart Arrest"[Mesh] OR "Heart Arrest, Induced"[Mesh] OR "Out-of-Hospital Cardiac Arrest"[Mesh]) AND ((clinicaltrial[Filter] OR meta-analysis[Filter] OR randomizedcontrolledtrial[Filter] OR review[Filter] OR systematicreview[Filter]) AND (english[Filter]))

("Community Health Workers"[Mesh] OR "community health representative" OR "community outreach worker" OR "community educator" OR "community care coordinator" OR "community health aide") AND ("Heart Failure"[Mesh] OR "CHF") AND ((clinicaltrial[Filter] OR meta-analysis[Filter] OR randomizedcontrolledtrial[Filter] OR review[Filter] OR systematicreview[Filter]) AND (english[Filter]))

("Stroke"[Mesh] OR "Ischemic Stroke"[Mesh] OR "Stroke Rehabilitation"[Mesh] OR "Stroke, Lacunar"[Mesh] OR "cerebrovascular incident") AND ("Community Health Workers"[Mesh] OR "community health representative" OR "community outreach worker" OR "community educator" OR "community care coordinator" OR "community health aide") AND ((clinicaltrial[Filter] OR meta-analysis[Filter] OR randomizedcontrolledtrial[Filter] OR review[Filter] OR systematicreview[Filter]) AND (english[Filter]))

("Hyperlipidemias[Mesh]" OR "HLD") AND("Community Health Workers"[Mesh] OR "community health representative" OR "community outreach worker" OR "community educator" OR "community care coordinator" OR "community health aide") AND ((clinicaltrial[Filter] OR meta-analysis[Filter] OR randomizedcontrolledtrial[Filter] OR review[Filter] OR systematicreview[Filter]) AND (english[Filter]))

("Hypertension"[Mesh] OR "HTN") AND ("Community Health Workers"[Mesh] OR "community health representative" OR "community outreach worker" OR "community educator" OR "community care coordinator" OR "community health aide") AND ((clinicaltrial[Filter] OR meta-analysis[Filter] OR randomizedcontrolledtrial[Filter] OR review[Filter] OR systematicreview[Filter]) AND (english[Filter]))

**SUPPL_3_Preferred Reporting Items for Systematic reviews and Meta-Analyses extension for Scoping Reviews (PRISMA-ScR) Checklist**

| **SECTION** | **ITEM** | **PRISMA-ScR CHECKLIST ITEM** | **REPORTED ON PAGE #** |
| --- | --- | --- | --- |
| **TITLE** | | | |
| Title | 1 | Identify the report as a scoping review. | Title Page |
| **ABSTRACT** | | | |
| Structured summary | 2 | Provide a structured summary that includes (as applicable): background, objectives, eligibility criteria, sources of evidence, charting methods, results, and conclusions that relate to the review questions and objectives. | Abstract |
| **INTRODUCTION** | | | |
| Rationale | 3 | Describe the rationale for the review in the context of what is already known. Explain why the review questions/objectives lend themselves to a scoping review approach. | Introduction/Methods |
| Objectives | 4 | Provide an explicit statement of the questions and objectives being addressed with reference to their key elements (e.g., population or participants, concepts, and context) or other relevant key elements used to conceptualize the review questions and/or objectives. | Introduction |
| **METHODS** | | | |
| Protocol and registration | 5 | Indicate whether a review protocol exists; state if and where it can be accessed (e.g., a Web address); and if available, provide registration information, including the registration number. | N/A |
| Eligibility criteria | 6 | Specify characteristics of the sources of evidence used as eligibility criteria (e.g., years considered, language, and publication status), and provide a rationale. | Methods |
| Information sources* | 7 | Describe all information sources in the search (e.g., databases with dates of coverage and contact with authors to identify additional sources), as well as the date the most recent search was executed. | Methods |
| Search | 8 | Present the full electronic search strategy for at least 1 database, including any limits used, such that it could be repeated. | Methods/Supplemental |
| Selection of sources of evidence† | 9 | State the process for selecting sources of evidence (i.e., screening and eligibility) included in the scoping review. | Methods |
| Data charting process‡ | 10 | Describe the methods of charting data from the included sources of evidence (e.g., calibrated forms or forms that have been tested by the team before their use, and whether data charting was done independently or in duplicate) and any processes for obtaining and confirming data from investigators. | Methods |
| Data items | 11 | List and define all variables for which data were sought and any assumptions and simplifications made. | Methods |
| Critical appraisal of individual sources of evidence§ | 12 | If done, provide a rationale for conducting a critical appraisal of included sources of evidence; describe the methods used and how this information was used in any data synthesis (if appropriate). | Not Reported |
| Synthesis of results | 13 | Describe the methods of handling and summarizing the data that were charted. | Supplemental |
| **RESULTS** | | | |
| Selection of sources of evidence | 14 | Give numbers of sources of evidence screened, assessed for eligibility, and included in the review, with reasons for exclusions at each stage, ideally using a flow diagram. | Methods |
| Characteristics of sources of evidence | 15 | For each source of evidence, present characteristics for which data were charted and provide the citations. | Results |
| Critical appraisal within sources of evidence | 16 | If done, present data on critical appraisal of included sources of evidence (see item 12). | Not Reported |
| Results of individual sources of evidence | 17 | For each included source of evidence, present the relevant data that were charted that relate to the review questions and objectives. | Results/ Supplemental |
| Synthesis of results | 18 | Summarize and/or present the charting results as they relate to the review questions and objectives. | Results/Discussion |
| **DISCUSSION** | | | |
| Summary of evidence | 19 | Summarize the main results (including an overview of concepts, themes, and types of evidence available), link to the review questions and objectives, and consider the relevance to key groups. | Discussion/Conclusion |
| Limitations | 20 | Discuss the limitations of the scoping review process. | Discussion |
| Conclusions | 21 | Provide a general interpretation of the results with respect to the review questions and objectives, as well as potential implications and/or next steps. | Conclusion |
| **FUNDING** | | | |
| Funding | 22 | Describe sources of funding for the included sources of evidence, as well as sources of funding for the scoping review. Describe the role of the funders of the scoping review. | Funding/ Conflict of Interest |

JBI = Joanna Briggs Institute; PRISMA-ScR = Preferred Reporting Items for Systematic reviews and Meta-Analyses extension for Scoping Reviews.

* Where *sources of evidence* (see second footnote) are compiled from, such as bibliographic databases, social media platforms, and Web sites.

† A more inclusive/heterogeneous term used to account for the different types of evidence or data sources (e.g., quantitative and/or qualitative research, expert opinion, and policy documents) that may be eligible in a scoping review as opposed to only studies. This is not to be confused with *information sources* (see first footnote).

‡ The frameworks by Arksey and O’Malley (6) and Levac and colleagues (7) and the JBI guidance (4, 5) refer to the process of data extraction in a scoping review as data charting*.*

§ The process of systematically examining research evidence to assess its validity, results, and relevance before using it to inform a decision. This term is used for items 12 and 19 instead of "risk of bias" (which is more applicable to systematic reviews of interventions) to include and acknowledge the various sources of evidence that may be used in a scoping review (e.g., quantitative and/or qualitative research, expert opinion, and policy document).

*From:* Tricco AC, Lillie E, Zarin W, O'Brien KK, Colquhoun H, Levac D, et al. PRISMA Extension for Scoping Reviews (PRISMAScR): Checklist and Explanation. Ann Intern Med. 2018;169:467–473. [doi: 10.7326/M18-0850](http://annals.org/aim/fullarticle/2700389/prisma-extension-scoping-reviews-prisma-scr-checklist-explanation).
